# High Prolyl 4-Hydroxylase Subunit Alpha 3 Expression as an Independent Prognostic Biomarker and Correlated With Immune Infiltration in Gastric Cancer

**DOI:** 10.3389/fgene.2022.952335

**Published:** 2022-07-01

**Authors:** Xiaoji Niu, Liman Ren, Shoumei Wang, Dong Gao, Mingyue Ma, Aiyan Hu, Hongjun Qi, Shuhui Zhang

**Affiliations:** ^1^ Department of Gastroenterology of Traditional Chinese Medicine, Qinghai Province Hospital of Traditional Chinese Medicine, Xining, China; ^2^ Department of Pathology, Yueyang Hospital of Integrated Traditional Chinese and Western Medicine, Shanghai University of Traditional Chinese Medicine, Shanghai, China; ^3^ Department of Endocrinology, Qinghai Province Hospital of Traditional Chinese Medicine, Xining, China

**Keywords:** P4HA3, gastric cancer, bioinformatics, prognosis, immune infiltration

## Abstract

**Background:** Gastric cancer (GC) has a high mortality rate and is particularly prevalent in China. The extracellular matrix protein, prolyl 4-hydroxylase subunit alpha 3 (P4HA3), has been implicated in various cancers. We aimed to assess the diagnostic and prognostic value of P4HA3 in GC and investigate its correlation with immune cell infiltration.

**Methods:** The present study used microarray data from the Cancer Genome Atlas (TCGA) to analyze the association of P4HA3 expression with clinicopathological features. Data from the Gene Expression Omnibus (GEO) were used for validation. Receiver operating characteristic (ROC) and Kaplan–Meier curves were constructed to determine the diagnostic and prognostic value of P4HA3 in GC. Univariate and multivariate regression analyses were performed to assess the impact of P4HA3 on overall survival (OS) rates. A protein–protein interaction (PPI) network was generated and functional enrichment evaluated. Single-sample gene set enrichment analysis (ssGSEA) was conducted to correlate P4HA3 expression with immune cell infiltration. The correlation between P4HA3 and immune check point genes was studied.

**Results:** P4HA3 was over-expressed in GC, along with 15 other types of cancer, including breast invasive carcinoma and cholangiocarcinoma. P4HA3 showed high diagnostic and prognostic value in GC and was an independent prognostic factor. P4HA3, TNM (tumor, node, metastases) stage, pathological stage and age all correlated with OS rates. Genes related to P4HA3 were enriched in the lumen of the endoplasmic reticulum and included procollagen-proline 3-dioxygenase activity. P4HA3 expression correlated with numbers of macrophages, natural killer (NK) cells, immature dendritic cells (iDC), mast cells, eosinophils, effective memory T cells (Tem), T-helper 1 (Th1) cells and dendritic cells (DC). P4HA3 was positively correlated with hepatitis A virus cellular receptor 2 (HAVCR2) and programmed cell death 1 ligand 2 (PDCD1LG2).

**Conclusion:** P4HA3 is a potential independent biomarker for prognosis of GC and may be an immunotherapy target in the treatment of GC.

## Introduction

Data from Global Cancer Statistics (https://gco.iarc.fr/today/online-analysis) indicates that gastric cancer (GC) is the fifth most frequently diagnosed cancer and the fourth leading cause of cancer-related death worldwide. Global age-standardized incidence and mortality rates are 11.1 per 100,000 and 7.7 per 100,000. Rates are considerably higher in China, where incidence and mortality occur at 20.6 per 100,000 and 15.9 per 100,000, respectively (Machlowska et al., 2020; Sung et al., 2021; Niu et al., 2022). GC is thus a significant health and economic burden worldwide and this is particularly the case in China.

The majority of GC cases are diagnosed at the late stage, resulting in a poor prognosis. However, advances in molecular biology techniques allow us to approach an understanding of precise molecular mechanisms of carcinogenesis which holds promise for development of diagnostic, prognostic and therapeutic strategies. It is known that immune-related mechanisms and markers participate in the occurrence and development of GC and appropriately targeted therapy looks promising for its treatment (Güthle et al., 2020). Such observations highlight the urgent need to identify new immune-related biomarkers to facilitate early GC diagnosis and treatment.

Prolyl 4-hydroxylase subunit alpha 3 (P4HA3) is a catalytic subunit involved in collagen synthesis. Its overexpression has been associated with tumors and with non-cancerous diseases, including idiopathic pituitary adenoma, melanoma, stomach carcinoma, breast cancer and pulmonary fibrosis (Luo et al., 2015; Song et al., 2018; Atkinson et al., 2019; Long et al., 2019; Gu et al., 2020).

A recent study has suggested that upregulation of P4HA3 is associated with enhanced metastasis and poor survival of GC patients (Song et al., 2018). However, any correlation with immune cell infiltration has been little scrutinized. The current study investigated P4HA3 expression in GC and its relationship with immune cell infiltration.

## Material and Methods

### Microarray Datasets

The Cancer Genome Atlas (TCGA) project (https://www.cancer.gov/tcga) is an open database which aims to make molecular data characterizing the cancer-related genome freely available and to link genomic data to patients’ clinicopathological information. RNA-sequencing data (level 3) with corresponding clinicopathological information were downloaded from the TCGA database. Data was converted from fragments per kilobase per million (FPKM) to transcripts per million reads (TPM). Survival data were published in Cell (Liu et al., 2018). For analyses across many tumor types, TCGA and Genotype-Tissue Expression Project (GTEx), TPM-formatted RNAseq data processed by Toil were downloaded from University of California Santa Cruz (UCSC) XENA (https://xenabrowser.net/datapages/) (Vivian et al., 2017). GSE54129 and GSE103236 datasets were obtained from the Gene Expression Omnibus (GEO) database (https://www.ncbi.nlm.nih.gov/geo/) to validate the P4HA3 expression level. Table 1 shows the details of expression datasets involved in the study.

**TABLE 1:** Details of Expression Datasets for the study.

Data source	ID	Platform	Samples (cancer vs. Normal)
TCGA	-	-	375 vs. 32
GEO	GSE54129	GPL570	111 vs. 21
GEO	GSE103236	GPL4133	10 vs. 9

### Diagnostic and Prognostic Value of P4HA3

Receiver operating characteristic (ROC) and Kaplan-Meier survival curves were constructed and used to analyze the diagnostic and prognostic value of P4HA3, respectively. The association between P4HA3 expression and overall survival (OS) rates of GC patients was assessed by univariate and multivariate regression analyses.

### PPI Network Construction and Functional Enrichment Analysis

Protein–protein interaction (PPI) network analysis of P4HA3 was performed by using the search tool of a single named protein with default parameters within the STRING database (version 11.5, accessed date: 02 June 2022) (Szklarczyk et al., 2021). Pathway and process enrichment analysis were conducted with the following ontology sources: Gene Ontology (GO) Biological Processes, GO Cellular Components, GO Molecular Functions, Kyoto Encyclopedia of Genes and Genomes (KEGG) Pathway, Reactome Gene Sets and Canonical Pathways within the Metascape database (https://metascape.org/gp/index.html) (Hochberg and Benjamini, 1990; Zhou et al., 2019). The complete proteome were regarded as the background to enrichment. Terms with a *p*-value less than 0.05, a minimum count of 3 and an enrichment factor (ratio of observed counts: counts expected by chance) of more than 1.5 were acquired and grouped into clusters based on their connections. *p*-values were calculated from the cumulative hypergeometric distribution and q-values using the Banjamini-Hochberg procedure to account for multiple testing. Kappa scores were used as the similarity metric when performing hierachical clustering of the enriched terms and sub-trees with a similarity of >0.3 were considered a cluster. The most statistically significant term within a cluster was used to represent that cluster.

### Correlation Analysis of Immune Cell Infiltration

ssGSEA was used to determine relationships between P4HA3 expression and 24 kinds of immune cells, including activated DC, B cells, macrophages and mast cells (Bindea et al., 2013). Spearman correlation analysis was used to evaluate the correlation between P4HA3 and immune cell infiltration and values of r > 0.3 or r < −0.3 and *p* < 0.05 were considered to indicate significant positive or negative correlation. The expression of the following immune-checkpoint–relevant transcripts was assessed: sialic acid binding Ig like lectin 15 (SIGLEC15), T cell immunoreceptor with Ig and ITIM domains (TIGIT), CD274 Molecule (CD274), hepatitis A virus cellular receptor 2 (HAVCR2), programmed cell death 1 (PDCD1), cytotoxic T-lymphocyte associated protein 4 (CTLA4), lymphocyte activating 3 (LAG3) and programmed cell death 1 ligand 2 (PDCD1LG2) (Zeng et al., 2019).

### Statistical Analysis

All the analytical methods (excluding functional enrichment analysis) were performed using Xiantao Academic (https://www.xiantao.love/products) embedded with R software and R packages, including org.Hs.eg.db, GEOquery, limma, ggplot2, clusterProfiler, survminer, survival and pROC (Davis and Meltzer, 2007; Yu et al., 2012; Liu et al., 2018; Hu et al., 2020). Chi-square test, paired *t* test and the Wilcoxon rank sum test were used to compare data. A value of *p* value < 0.05 was regarded as statistically significant.

## Results

### Clinicopathological Characteristics

**FIGURE 1:**
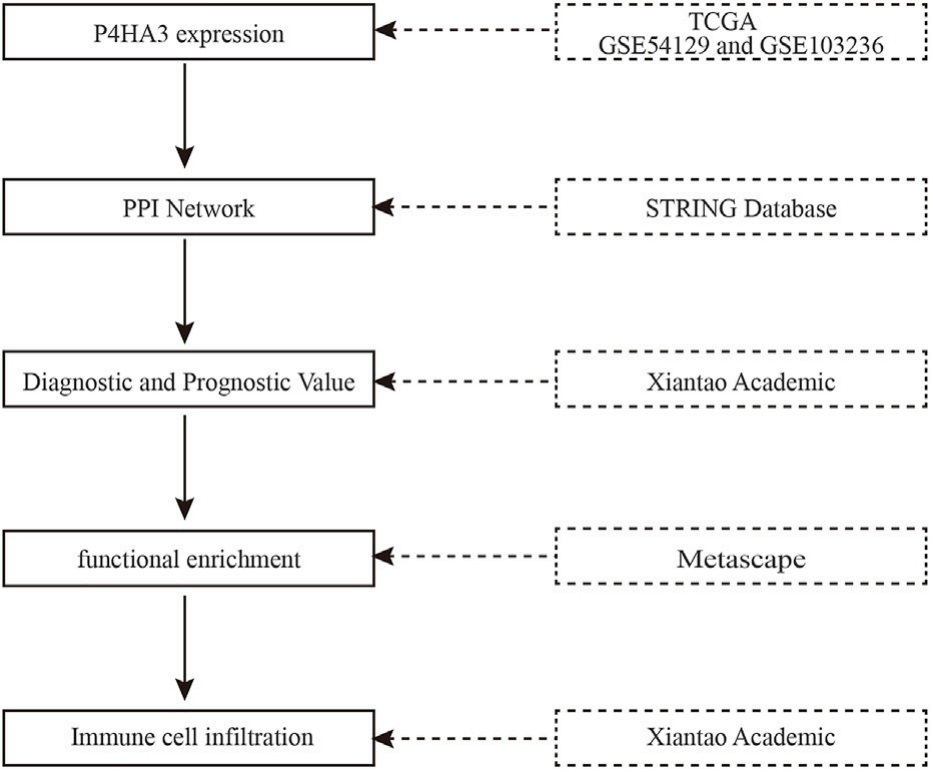
The flowchart of the present study.

The flowchart of the present study is presented in Figure 1. RNA-seq expression data from 624 samples, including 174 normal tissues, 36 para carcinoma tissues and 414 tumor tissues plus clinical data were downloaded from UCSC XENA. The details are presented in Table 2.

**TABLE 2:** Clinical characteristics of the GC patients based on TCGA.

Characteristic	Low expression of P4HA3	High expression of P4HA3	*p*
n	187	188	
T stage, n (%)			0.003
T1	17 (4.6%)	2 (0.5%)	
T2	41 (11.2%)	39 (10.6%)	
T3	85 (23.2%)	83 (22.6%)	
T4	43 (11.7%)	57 (15.5%)	
N stage, n (%)			0.885
N0	55 (15.4%)	56 (15.7%)	
N1	51 (14.3%)	46 (12.9%)	
N2	35 (9.8%)	40 (11.2%)	
N3	38 (10.6%)	36 (10.1%)	
M stage, n (%)			0.988
M0	166 (46.8%)	164 (46.2%)	
M1	12 (3.4%)	13 (3.7%)	
Pathologic stage, n (%)			0.154
Stage I	34 (9.7%)	19 (5.4%)	
Stage II	50 (14.2%)	61 (17.3%)	
Stage III	76 (21.6%)	74 (21%)	
Stage IV	19 (5.4%)	19 (5.4%)	
Primary therapy outcome, n (%)			0.099
PD	38 (12%)	27 (8.5%)	
SD	7 (2.2%)	10 (3.2%)	
PR	0 (0%)	4 (1.3%)	
CR	116 (36.6%)	115 (36.3%)	
Gender, n (%)			0.564
Female	70 (18.7%)	64 (17.1%)	
Male	117 (31.2%)	124 (33.1%)	
Age, n (%)			0.437
≤65	86 (23.2%)	78 (21%)	
>65	99 (26.7%)	108 (29.1%)	
Histological type, n (%)			0.005
Diffuse Type	27 (7.2%)	36 (9.6%)	
Mucinous Type	7 (1.9%)	12 (3.2%)	
Not Otherwise Specified	97 (25.9%)	110 (29.4%)	
Papillary Type	3 (0.8%)	2 (0.5%)	
Signet Ring Type	4 (1.1%)	7 (1.9%)	
Tubular Type	49 (13.1%)	20 (5.3%)	
Histologic grade, n (%)			0.009
G1	5 (1.4%)	5 (1.4%)	
G2	82 (22.4%)	55 (15%)	
G3	95 (26%)	124 (33.9%)	
*H pylori* infection, n (%)			0.869
No	88 (54%)	57 (35%)	
Yes	10 (6.1%)	8 (4.9%)	
Barretts esophagus, n (%)			0.461
No	116 (55.8%)	77 (37%)	
Yes	11 (5.3%)	4 (1.9%)	

Abbreviations: CR, complete response; PD, progressive disease; SD, stable disease; PR, partial response.

### Expression of P4HA3 Across Many Cancer Cell-Types

**FIGURE 2:**
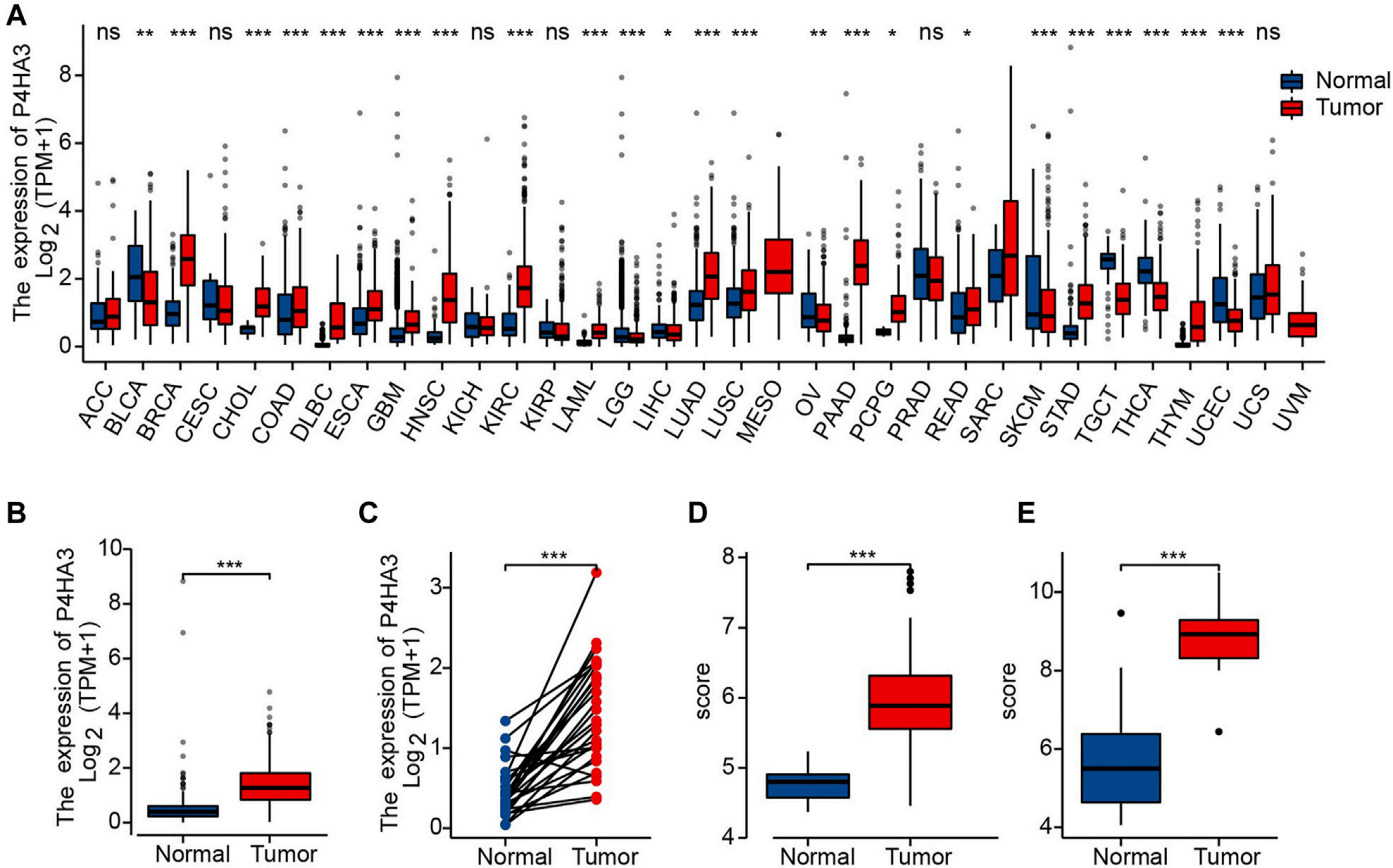
Upregulation of P4HA3 in GC. **(A)** P4HA3 expression levels in various cancer-types from TCGA data; **(B)** P4HA3 transcript levels in GC and non-cancerous gastric tissues from TCGA data; **(C)** P4HA3 expression in paired samples; **(D)** P4HA3 expression in GSE54129; **(E)** P4HA3 expression in GSE103236. (ns, *p* ≥ 0.05; *, *p* < 0.05; **, *p* < 0.01; ***, *p* < 0.001.) Abbreviations: P4HA3, prolyl 4-hydroxylase subunit alpha 3; GC, gastric cancer; BRCA, breast invasive carcinoma; CHOL, cholangiocarcinoma; COAD, colon adenocarcinoma; DLBC, lymphoid neoplasm diffuse large B-cell lymphoma; ESCA, esophageal carcinoma; GBM, glioblastoma multiforme, HNSC, head and neck squamous cell carcinoma; KIRC, kidney renal clear cell carcinoma; LAML, acute myeloid leukemia; LUAD, lung adenocarcinoma; LUSC, lung squamous cell carcinoma; PAAD, pancreatic adenocarcinoma; PCPG, pheochromocytoma and paraganglioma; READ, rectum adenocarcinoma; THYM, thymoma; STAD, stomach adenocarcinoma; THCA, thyroid carcinoma.

Differential expression of P4HA3 mRNA was measured and found to be over-expressed in 16 cancers, including breast invasive carcinoma (BRCA), cholangiocarcinoma (CHOL), colon adenocarcinoma (COAD), lymphoid neoplasm diffuse large B-cell lymphoma (DLBC), esophageal carcinoma (ESCA), glioblastoma multiforme (GBM), head and neck squamous cell carcinoma (HNSC), kidney renal clear cell carcinoma (KIRC), acute myeloid leukemia (LAML), lung adenocarcinoma (LUAD), lung squamous cell carcinoma (LUSC), pancreatic adenocarcinoma (PAAD), pheochromocytoma and paraganglioma (PCPG), rectum adenocarcinoma (READ), thymoma (THYM) and stomach adenocarcinoma (STAD; Figure 2A). All-sample and paired sample analysis showed that expression levels of P4HA3 were higher in GC tissue than in non-cancerous tissue (Figures 2B,C). Further analysis using GSE54129 (Figure 2D) and GSE103236 (Figure 2E) gave similar results to those from the TCGA data.

### P4HA3 Expression and GC Clinicopathological Features

**FIGURE 3:**
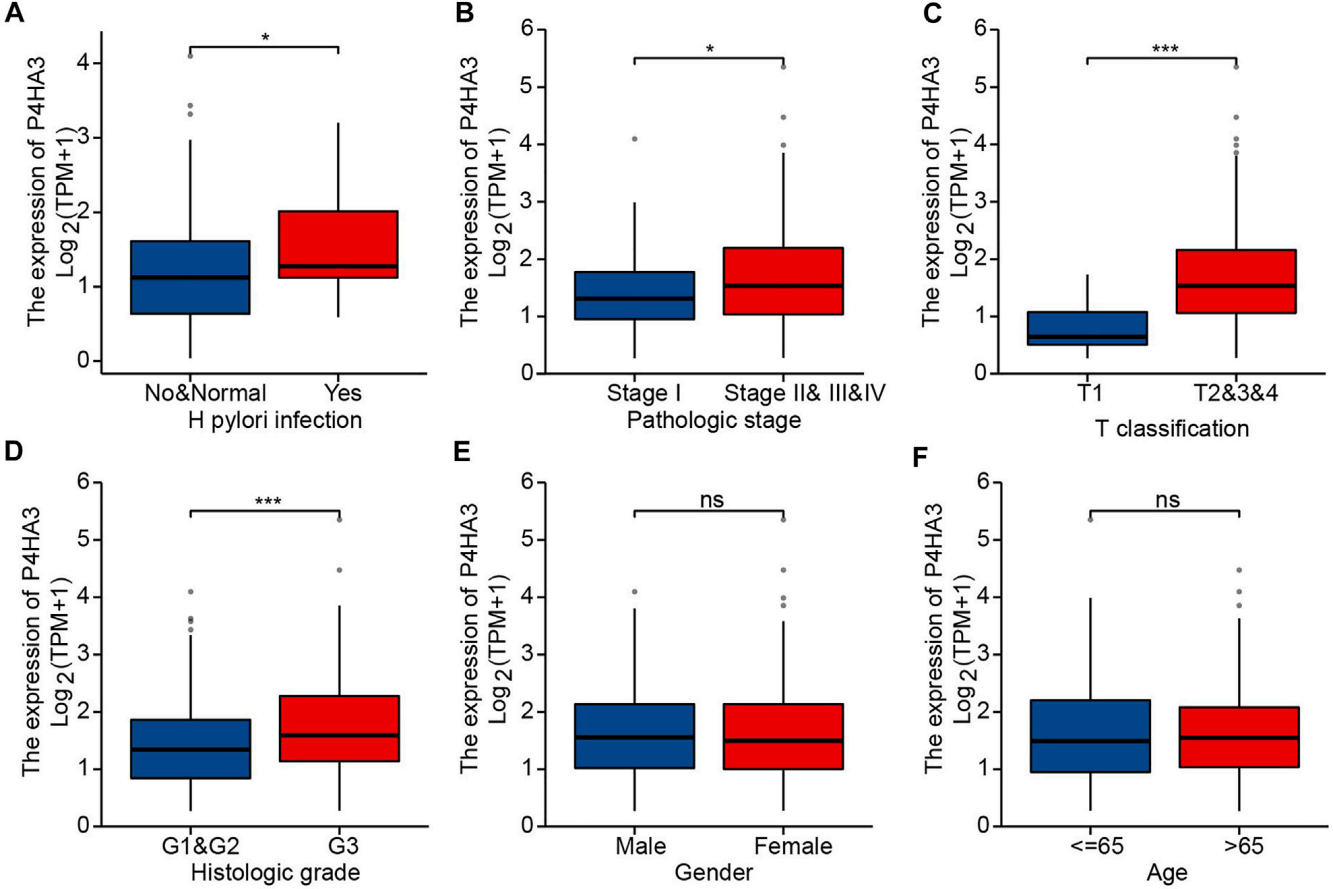
P4HA3 expression is associated with clinicopathological characteristics. **(A)** Post-mortal P4HA3 expression levels were higher; **(B)** Late stage P4HA3 expression levels were higher; **(C)** P4HA3 expression levels were higher in patients with higher T classifications; **(D)** P4HA3 expression levels were higher in patients with lower histological grades; **(E)** No gender differences were found for P4HA3 expression levels; **(F)** No age-related changes were found in P4HA3 expression levels. ns, *p* ≥ 0.05; *, *p* < 0.05; ***, *p* < 0.001.

The correlation analysis showed significant differences for some clinicopathological features, including *Helicobacter pylori* infection status (Figure 3A), pathological stage (Figure 3B), T classification (Figure 3C) and histological grade (Figure 3D). There were no differences in P4HA3 expression based on gender (Figure 3E) or age (Figure 3F).

### Correlation Analysis of Prognosis

**FIGURE 4:**
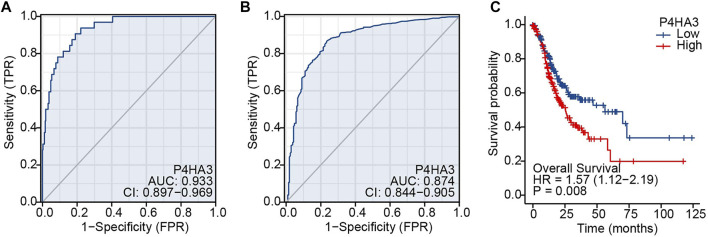
ROC and Kaplan-Meier survival curves. **(A)** ROC curve for P4HA3 based on data from TCGA; **(B)** ROC curve for P4HA3 using data from non-cancerous samples from GTEx combined para carcinoma tissues and GC samples; **(C)** Higher levels of P4HA3 expression tended to be associated with worse outcomes (OS) for GC patients. Abbreviations: ROC, receiver operating characteristic; GTEx, Genotype-Tissue Expression Project.

The area under the ROC curve was 0.933, based on TCGA data (Figure 4A), and 0.874 for non-cancerous samples of GTEx combined para carcinoma tissues and GC samples (Figure 4B). These results indicate that levels of P4HA3 expression are consistently different in tumor and non-tumor tissues. Kaplan-Meier survival analysis indicated that high levels of P4HA3 are associated with poor prognosis (Figure 4C).

Univariate analysis demonstrated that high P4HA3 expression corresponded to reduced OS and, thus, poor prognosis for GC patients (Table 3). TNM stage, pathological stage and age were also associated with reduced OS (Table 3). The results of multivariate analysis showed that P4HA3 was an independent prognostic marker. TNM stage and age also had independent prognostic value for OS in GC (Table 3).

**TABLE 3:** Univariate and multivariate Cox regression analysis of the P4HA3 expression and overall survival in gastric cancer patients.

Variable	Total (*N*)	Univariate analysis		Multivariable	
		HR (95% CI)	*p* value	HR (95% CI)	*p* value
T stage (T1 vs. T2&3&4)	362	8.829 (1.234-63.151)	**0.030**	3.735 (0.502-27.792)	0.198
N stage (N0 vs. N1&2&3)	352	1.925 (1.264-2.931)	**0.002**	1.356 (0.749-2.454)	0.314
M stage (M0 vs. M1)	352	2.254 (1.295-3.924)	**0.004**	1.959 (1.015-3.781)	**0.045**
Pathological stage (Stage I& II vs. III& IV)	347	1.947 (1.358-2.793)	**<0.001**	1.371 (0.819-2.294)	0.230
Grade (G1&G2 vs. G3)	361	1.353 (0.957-1.914)	0.087	1.290 (0.878-1.894)	0.194
Age (≤65 vs. >65)	367	1.620 (1.154-2.276)	**0.005**	1.866 (1.278-2.725)	**0.001**
Gender (Female vs. Male)	370	1.267 (0.891-1.804)	0.188	-	-
*H pylori* infection (NO vs. Yes)	162	0.650 (0.279-1.513)	0.317	-	-
Reflux history (No vs. Yes)	213	0.582 (0.291-1.162)	0.125	-	-
P4HA3 (Low vs. High)	370	1.634 (1.169-2.284)	**0.004**	1.641 (1.135-2.374)	**0.008**

Notes: Bold type indicates statistical significance.

Abbreviations: P4HA3, Prolyl 4-hydroxylase subunit alpha 3; HR, hazard ratio; CI: confidence interval.

### PPI Networks and Enrichment Analysis

**FIGURE 5:**
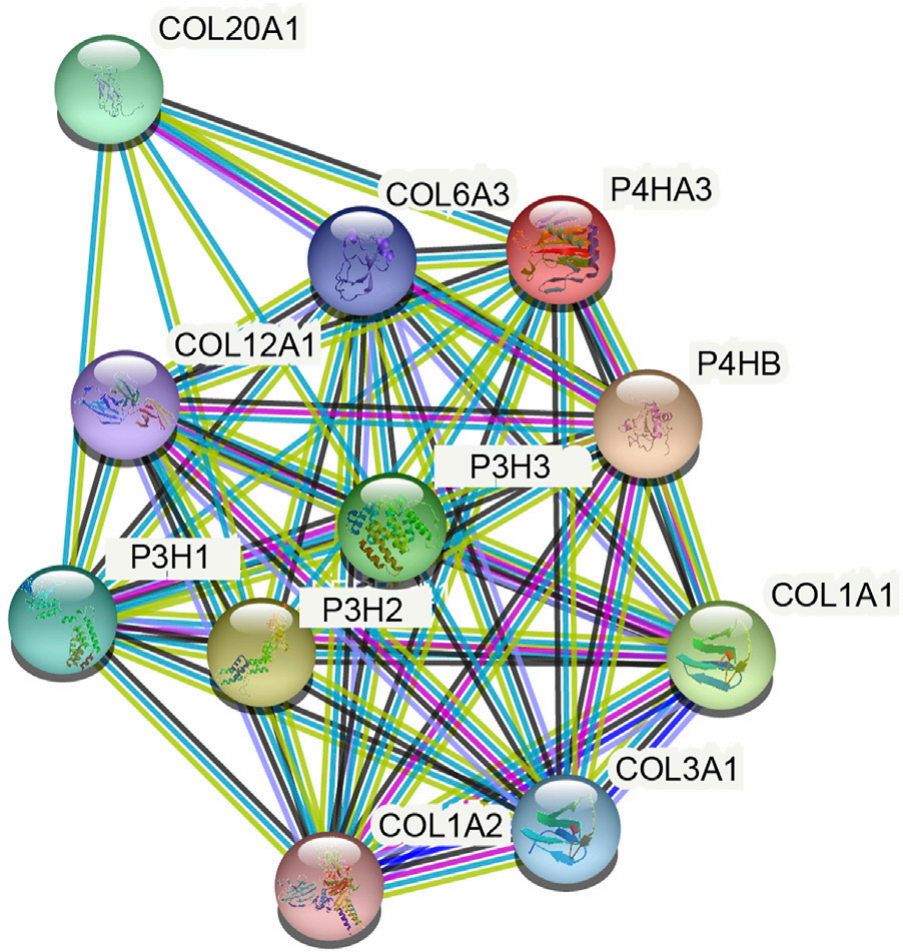
Protein-protein interaction (PPI) network of P4HA3-related proteins (TOP 10, medium confidence).

The present study reports the construction of a network of P4HA3 and its related genes using the STRING database. P4HA3-related genes with scores above 0.9 included Collagen Type I Alpha (COL1A)1, COL1A2, COL3A1, COL6A3, COL12A1, COL20A1, prolyl 3-hydroxylase (P3H)1, P3H2 and P3H3 (Figure 5; Table 4). Metascape pathway and process enrichment analysis revealed that all genes related to P4HA3 were enriched in R-HSA-1650814, suggesting roles in collagen biosynthesis and modifying enzymes. The endoplasmic reticulum lumen and procollagen-proline 3-dioxygenase activity were also associated with P4HA3 (Table 5).

**TABLE 4:** The detailed information of P4HA3-related genes.

Gene symbol	Annotation	Score
COL1A1	collagen type I alpha 1 chain	0.951
COL1A2	collagen type I alpha 2 chain	0.926
COL3A1	collagen type III alpha 1 chain	0.933
COL6A3	collagen type VI alpha 3 chain	0.927
COL12A1	collagen type XII alpha 1 chain	0.927
COL20A1	collagen type XX alpha 1 chain	0.941
P3H1	prolyl 3-hydroxylase 1	0.934
P3H2	prolyl 3-hydroxylase 2	0.959
P3H3	prolyl 3-hydroxylase 3	0.944

**TABLE 5:** Clusters with their representative enriched terms (one per cluster).

Term	Category	Description	Count (%)	*p*	q
R-HSA-1650814	Reactome Gene Sets	Collagen biosynthesis and modifying enzymes	9 (100)	<0.001	<0.001
M3005	Canonical Pathways	NABA COLLAGENS	6 (66.67)	<0.001	<0.001
GO:0005788	GO Cellular Components	endoplasmic reticulum lumen	8 (88.89)	<0.001	<0.001
GO:0019797	GO Molecular Functions	procollagen-proline 3-dioxygenase activity	3 (33.33)	<0.001	<0.001

Abbreviation: GO, gene ontology.

### Correlation Analysis of Immune Infiltration

**FIGURE 6:**
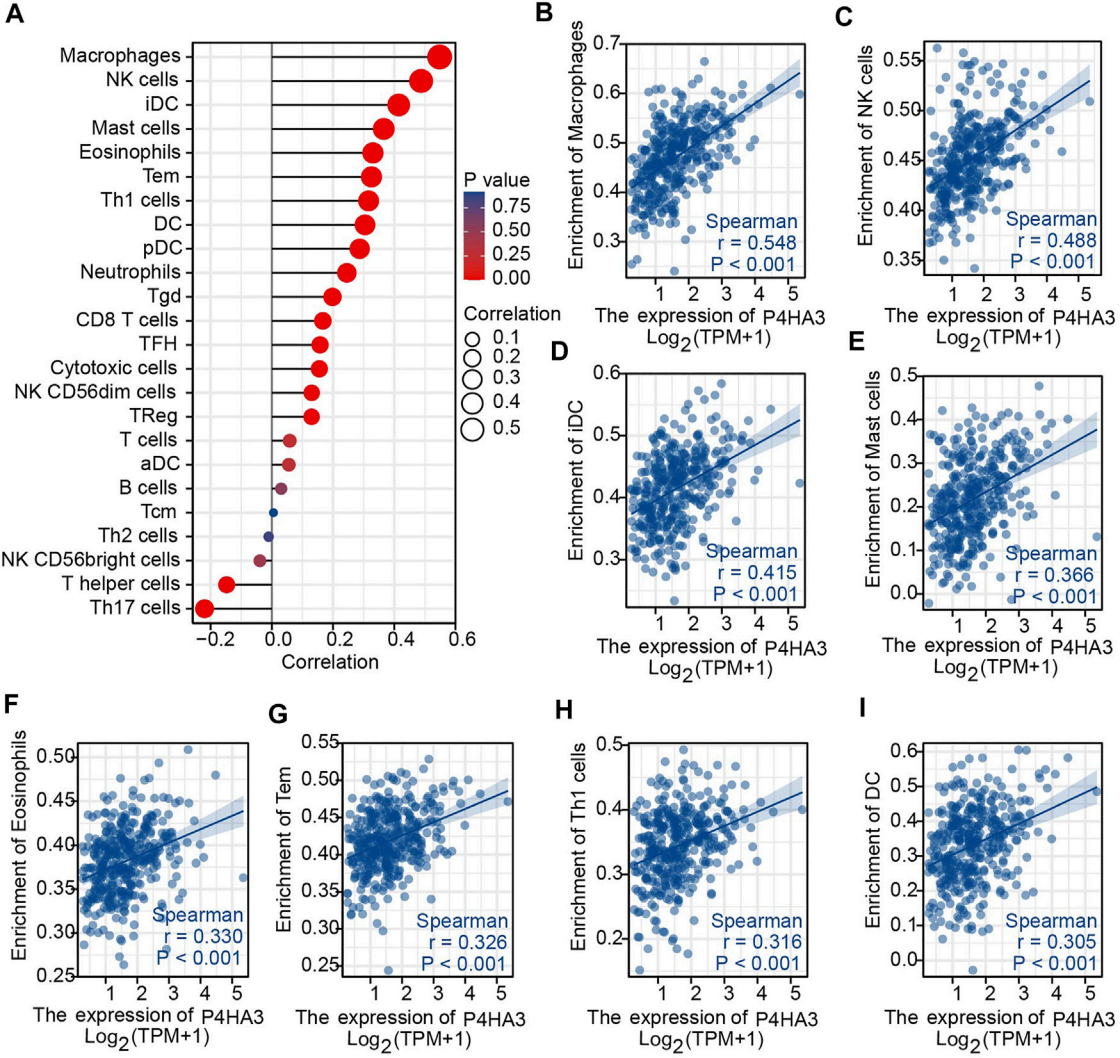
Immune Cell Infiltration Analysis. **(A)** The Lollipop Chart shows the correlation between P4HA3 expression level and 24 different immune cell-types; **(B–I)** the enrichment scores of P4HA3 expression for 8 immune cell-types. Abbreviations: NK, natural killer; iDC, immature dendritic cells; Tem, effective memory T cells; Th1, T-helper 1, DC, dendritic cells.

The association between P4HA3 and the degree of immune cell infiltration in GC was explored using ssGSEA analysis (Figure 6A). Macrophages, NK cells, iDC, mast cells, eosinophils, Tem, Th1 cells and DC all correlated with P4HA3 (Figures 6B–I).

**FIGURE 7:**
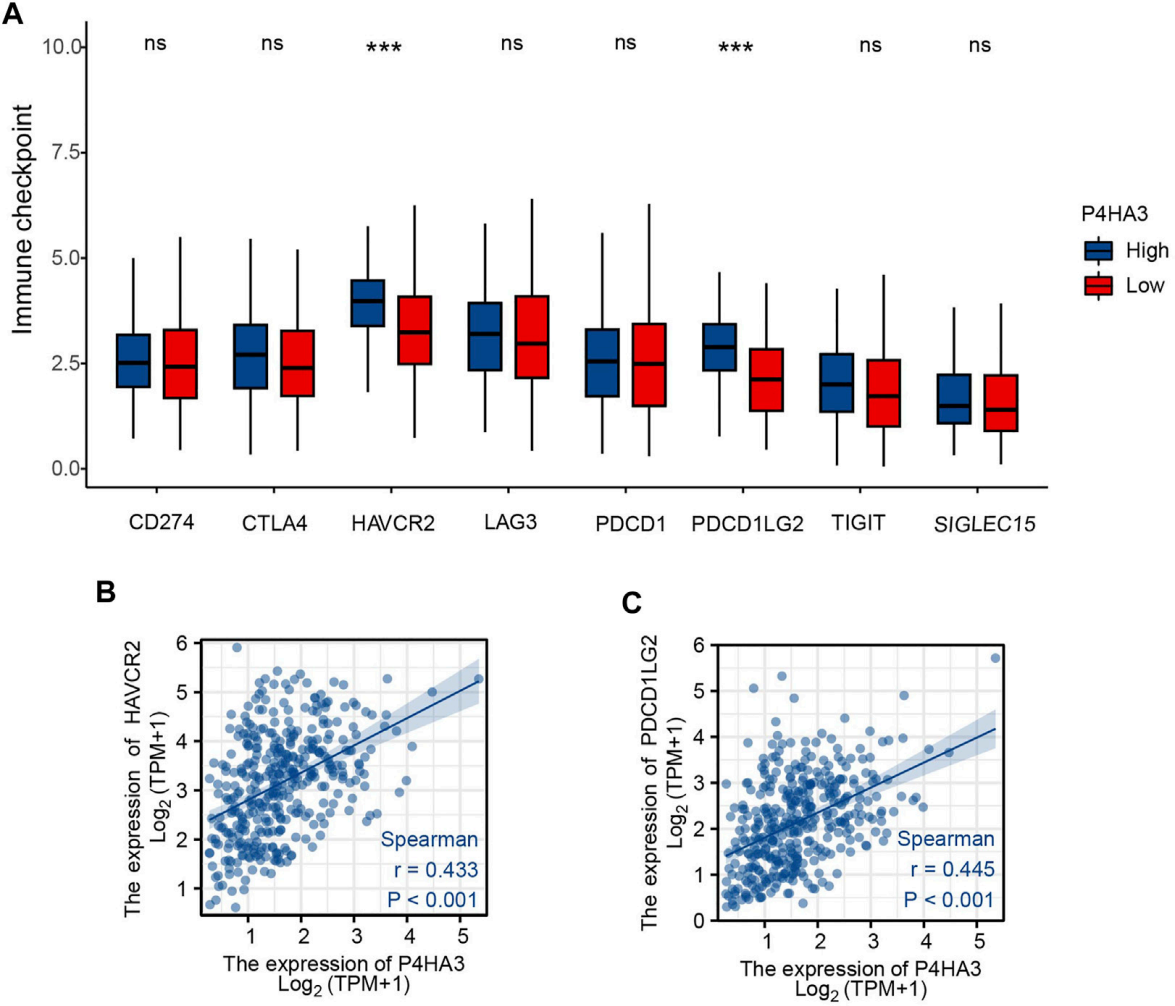
**(A)** Differential expression of immune-checkpoint–relevant genes in low and high P4HA3-expressing groups. **(B)** P4HA3 was positively correlated with HAVCR2. **(C)** P4HA3 was positively correlated with PDCD1LG2. ns, *p* > 0.05; ***, *p* < 0.001. Abbreviations: HAVCR2, hepatitis A virus cellular receptor 2; PDCD1LG2, programmed cell death 1 ligand 2.

Patients were divided into two groups according to P4HA3 expression and those with high expression had higher levels of the immune-checkpoint–relevant transcripts, HAVCR2 and PDCD1LG2, than those with low expression (Figure 7A). P4HA3 expression was positively correlated with HAVCR2 (Figure 7B) and PDCD1LG2 (Figure 7C).

## Discussion

Prolyl 4-hydroxylase (P4H) activity is essential for maintenance of the collagen triple helix and P4HAs (P4HA1, P4HA2, P4HA3) plus P4HB are highly expressed in numerous tumors where they may contribute to cancer progression. A number of inhibitors of P4HAS and P4HB have been shown to exert anti-tumor effects, suggesting that P4H is an achievable target for cancer therapy (Shi et al., 2021). Expression profiles and functional roles of P4HA3 in GC have rarely been studied. The current study focused on the diagnostic, prognostic and potential immune therapeutic target value of P4HA3 in GC.

mRNA expression levels across many different cancer types were analyzed using data from the TCGA database. P4HA3 mRNA was up-regulated in GC, along with BRCA, CHOL, COAD, DLBC, ESCA, GBM, HNSC, KIRC, LAML, LUAD, LUSC, PAAD, PCPG, READ and THYM, in agreement with the previous study of Hu et al., 2020. P4HA3 up-regulation was confirmed using GSE54129 and GSE103236 from the GEO database.

A key component of the current study was to address the diagnostic and prognostic value of P4HA3. GC patients tended to have higher P4HA3 mRNA expression when they were infected by HP, resulting in diagnosis with higher T stages and lower histological grades, or when they were diagnosed at late pathological stages. ROC analysis indicated differences in P4HA3 expression between tumor tissues and non-cancerous tissues. Kaplan–Meier survival analysis indicated that those GC patients with higher levels of P4HA3 tended to have shorter OS. Multivariate Cox analysis demonstrated that high levels of P4HA3 mRNA constituted an independent risk factor for OS and were associated with poor GC prognosis. Thus, we believe that P4HA3 could serve as a novel diagnostic and independent prognostic biomarker for GC patients.

P4HA3 has been shown to have an association with many different cancers. It promoted cell proliferation, invasion and migration in head and neck squamous cell carcinoma and melanoma cells (Atkinson et al., 2019; Wang et al., 2020) and reduced the anti-tumor activity of COL6A6 on growth and metastasis of AtT-20 and HP75 melanoma cells due to an action on PI3K-Akt signaling (Long et al., 2019). P4HA3 is known to be upregulated in clear cell renal carcinoma and patients with higher expression had worse outcomes, indicating a prognostic role for P4HA3 (Liu et al., 2020). Findings of the present and previous studies indicate that P4HA3 maintains the stability of newly synthesized collagens and remodels the extracellular matrix in GC (Shoulders and Raines, 2009; Gilkes et al., 2013, 2014).

Macrophages and NK cells influence the tumor microenvironment and tumor immunity (Lee et al., 2014; Sammarco et al., 2019; Gambardella et al., 2020). Infiltration of M2 macrophages promotes tumor cell escape and thus, numbers may reflect prognosis (Liu X. et al., 2021). Infiltration of NK cells has an impact on immunotherapy and targeting NK cells may improve the anti-tumor immune response (Yang et al., 2019; Bi et al., 2021). P4HA3 expression correlated with immune infiltration by macrophages and NK cells.

Immune checkpoint molecules regulate self-tolerance to prevent autoimmune reactions and minimize tissue damage by controlling the length and intensity of the immune response. Expression of checkpoint molecules acts to limit the immune and anti-tumor immune response, enabling escape of tumor cells (Galluzzi et al., 2020; Liu Y. et al., 2021). Patients with higher P4HA3 mRNA expression tended to have higher expression of the immune checkpoint related genes, PDCD1LG2 and HAVCR2. The current findings indicate the potential for targeting of P4HA3 during GC immunotherapy.

In conclusion, the purpose of the current study was to determine the diagnostic and prognostic value of P4HA3 and its correlation with immune cell infiltration in GC. P4HA3 emerges as a feasible diagnostic and prognostic biomarker and immunotherapy target. However, the current results are all derived from bioinformatics analysis and limited by the absence of experimental confirmation. Further clinical experiments are underway to verify the function of P4HA3 in GC.

## Data Availability

The original contributions presented in the study are included in the article/Supplementary Material, further inquiries can be directed to the corresponding authors.
